# Predicting respiratory failure for COVID-19 patients in Japan: a simple clinical score for evaluating the need for hospitalisation

**DOI:** 10.1017/S0950268821001837

**Published:** 2021-07-30

**Authors:** Gen Yamada, Kayoko Hayakawa, Nobuaki Matsunaga, Mari Terada, Setsuko Suzuki, Yusuke Asai, Hiroshi Ohtsu, Ako Toyoda, Koji Kitajima, Shinya Tsuzuki, Sho Saito, Norio Ohmagari

**Affiliations:** 1 Disease Control and Prevention Center, National Center for Global Health and Medicine, Tokyo, Japan; 2 AMR Clinical Reference Center, National Center for Global Health and Medicine, Tokyo, Japan; 3 Center for Clinical Sciences, National Center for Global Health and Medicine, Tokyo, Japan; 4 Faculty of Medicine and Health Sciences, University of Antwerp, Antwerp, Belgium

**Keywords:** COVID-19, prediction model, respiratory failure, risk scoring

## Abstract

Predicting the need for hospitalisation of patients with coronavirus disease 2019 (COVID-19) is important for preventing healthcare disruptions. This observational study aimed to use the COVID-19 Registry Japan (COVIREGI-JP) to develop a simple scoring system to predict respiratory failure due to COVID-19 using only underlying diseases and symptoms. A total of 6873 patients with COVID-19 admitted to Japanese medical institutions between 1 June 2020 and 2 December 2020 were included and divided into derivation and validation cohorts according to the date of admission. We used multivariable logistic regression analysis to create a simple risk score model, with respiratory failure as the outcome for young (18–39 years), middle-aged (40–64 years) and older (≥65 years) groups, using sex, age, body mass index, medical history and symptoms. The models selected for each age group were quite different. Areas under the receiver operating characteristic curves for the simple risk score model were 0.87, 0.79 and 0.80 for young, middle-aged and elderly derivation cohorts, and 0.81, 0.80 and 0.67 in the validation cohorts. Calibration of the model was good. The simple scoring system may be useful in the appropriate allocation of medical resources during the COVID-19 pandemic.

## Introduction

Coronavirus disease 2019 (COVID-19) shows a variety of clinical presentations. Most patients are asymptomatic or mildly ill at the time of diagnosis and then recover, but some require oxygen therapy, and more severe cases may require mechanical ventilation or may even die [1–3].

In Japan, COVID-19 patients were hospitalised in principle at the beginning of the pandemic [4]. However, as the number of patients requiring hospitalisation increased due to the spread of the infection and securing hospital beds for patients with severe disease became difficult, patients with mild disease were placed in facilities for recuperating (hotels provided by the government, etc.) or sent home. Even in mild cases, individuals who fell into one of the following categories were recommended for hospitalisation until at least 10 days after disease onset: (i) elderly individuals (≥65 years old); (ii) individuals with underlying diseases; (iii) individuals in an immunosuppressed state; (iv) individuals who were pregnant; and (v) individuals judged by doctors to require hospitalisation [5]. These hospitalisations have been coordinated by regional healthcare systems. Most hospitalised patients did not require oxygen therapy during the course of the disease [6], and many of these patients were discharged from the hospital after observation alone.

As of 25 March 2021, a cumulative total of 458 539 infections and 8936 deaths had been confirmed in Japan [7], and the number of patients has increased explosively since the second wave began in June 2020 [8]. In January 2021, securing inpatient beds became particularly difficult. In Tokyo, as the city with the largest number of patients, COVID-19 inpatients took up 84% of available beds (as of 13 January 2021) [9], and the number of patients requiring admission to an intensive care unit (ICU) was greater than the number of available ICU beds (113% as of 27 January 2021) [10].

From the perspective of efficient allocation of medical resources, in situations where the number of patients increases rapidly to the extent that securing inpatient beds is difficult, hospitalisation of patients with high oxygen demands (i.e. patients with a high possibility of developing respiratory failure) must be prioritised. In addition, in situations where patients are placed in recuperating facilities or sent home, and where detailed vital signs, physical findings and blood sampling data are unavailable, having an index for follow-up that focuses on patients with the highest possibility of developing respiratory failure is useful.

Most prognostic models for COVID-19 developed to date have focused on predicting death or ICU admission, and have required vital signs, blood sampling data and imaging studies [11]. These prediction models have been developed with small sample sizes, and relatively few models have predicted the need for oxygen therapy using large databases. This study aimed to develop and validate a simple clinical risk score to predict the need for oxygen therapy using only patient demographic characteristics, comorbidities and symptoms, based on a large registry of patients hospitalised for COVID-19 throughout Japan.

## Methods

This study is reported in accordance with Transparent Reporting of a multivariable prediction model for Individual Prognosis Or Diagnosis (TRIPOD) recommendations [12].

### Study population

This study was an observational study using the COVID-19 Registry Japan (COVIREGI-JP), a nationwide registry of patients hospitalised for COVID-19 [13]. Study data were collected and managed using REDCap (Research Electronic Data Capture), a secure, web-based data capture application hosted at the Joint Center for Researchers, Associates and Clinicians (JCRAC) data centre of the National Center for Global Health and Medicine (NCGM) [14]. Inclusion criteria for this study were as follows: (1) positive results for severe acute respiratory syndrome coronavirus 2 (SARS-CoV-2) from polymerase chain reaction (PCR) testing; and (2) hospitalisation in a Japanese medical institution between 1 June 2020 and 2 December 2020. Exclusion criteria were as follows: (1) >14 days from symptom onset to hospitalisation; (2) already hospitalised at the time of COVID-19 diagnosis; (3) transferred from the reporting hospital to another hospital; (4) age <18 years; (5) pregnant; (6) receiving haemodialysis; or (7) missing values for outcome. This study was conducted in accordance with the Declaration of Helsinki and was approved by the Ethics Review Committee at the NCGM (approval number: NCGM-G-003494-0). Information regarding opting out of this study is available on the registry website.

### Outcome and predictor variables

Primary outcome was the presence or absence of oxygen therapy (by any method) during hospitalisation. Predictor variables were selected as clinically important factors or those identified as risk factors for severe disease in previous studies [15–22], and data at the time of admission were used (Supplementary Table S1). The 19 candidate factors were demographic characteristics (age, sex, body mass index (BMI)), presence of symptoms (fever, cough, shortness of breath (SOB), wheezing, or fatigue) and presence of comorbidities (hypertension, hyperlipidaemia, diabetes mellitus, congestive heart failure, cerebrovascular disease, bronchial asthma, chronic lung disease other than bronchial asthma, chronic liver disease, moderate to severe renal dysfunction, collagen disease or malignant disease). According to the classification of BMI for Asians proposed by the World Health Organization [23, 24], BMI was classified into five categories: <18.5, 18.5–22.9, 23.0–24.9, 25.0–29.9 or ≥30 kg/m^2^.

### Statistical analysis

The study population was divided into two cohorts according to the date of admission: derivation cohort (from 1 June 2020 to 10 September 2020) and validation cohort (from 11 September 2020 to 2 December 2020) [12, 25]. Summary statistics of baseline characteristics at admission for each cohort were calculated and expressed appropriately as mean (standard deviation), median (interquartile range) or count (percentage), as appropriate.

#### Model development

According to previous studies, the proportion of young patients who required respiratory support was much lower than that of elderly patients [6], and some risk factors for severe disease were reported to be highly heterogeneous according to age [26]. In this study, we divided the study population into three age groups: young, 18–39 years old; middle-aged, 40–64 years old; and elderly, ≥65 years old [27, 28]. We then created a model for each age group. The study population was further categorised into the following age strata: 18–29, 30–39, 40–49, 50–59, 60–64, 65–74 and ≥75 years old.

All 19 predictor variables were entered into a logistic regression model with the presence or absence of oxygen administration during hospitalisation as the outcome. Variables were selected by the backward elimination method, and regression coefficients of each variable were used to create a comprehensive model [25, 29]. In addition, a simple risk score model was created by dividing all regression coefficients by the smallest of the binary predictor regression coefficients in the comprehensive model and converting values to the nearest integer [25].

#### Evaluation of performance

To evaluate the performance of the simple risk score model, areas under the receiver operating characteristic curves (AUCs) for discrimination were calculated separately for the derivation cohort and validation cohort. Sensitivity, specificity, positive predictive value (PPV) and negative predictive value (NPV) at various cut-off points were also calculated. Finally, to evaluate agreement between the predicted and observed probabilities, we created a calibration plot with the predicted probability on the *x*-axis and the observed probability on the *y*-axis using a comprehensive model.

#### Missing data handling and sensitivity analysis

Among the collected data on symptoms, those with entries of ‘unknown’ were treated as missing values, and missing data for symptoms were treated as ‘no symptoms’. For the 17.1% of patients with missing BMI data, the missing data were complemented using single imputation methods as follows. In primary analysis, missing BMI data were complemented by one of the five categories using multivariable logistic regression with the following predictors: age, sex, symptoms (fever, cough, SOB, wheezing and fatigue) and presence of comorbidities (hypertension, hyperlipidaemia, diabetes mellitus, congestive heart failure, cerebrovascular disease, bronchial asthma, chronic lung disease other than bronchial asthma, chronic liver disease, moderate to severe renal dysfunction, collagen disease and malignant disease). In sensitivity analyses, for patients with physician-diagnosed obesity according to registry data, BMI data were complemented by either one of two categories, 25.0–29.9 or ≥30 kg/m^2^. For patients without physician-diagnosed obesity, data were complemented by one of the five categories according to the distribution of observed BMIs.

Since this was an observational study, all available data were used in the analysis. All *P*-values were two-tailed, and the statistical significance level was set at the level of *P* < 0.05. All analyses were performed using SAS version 9.4 software (SAS Institute, Cary, NC, USA).

## Results

### Background characteristics of patients on admission

**Fig. 1.**
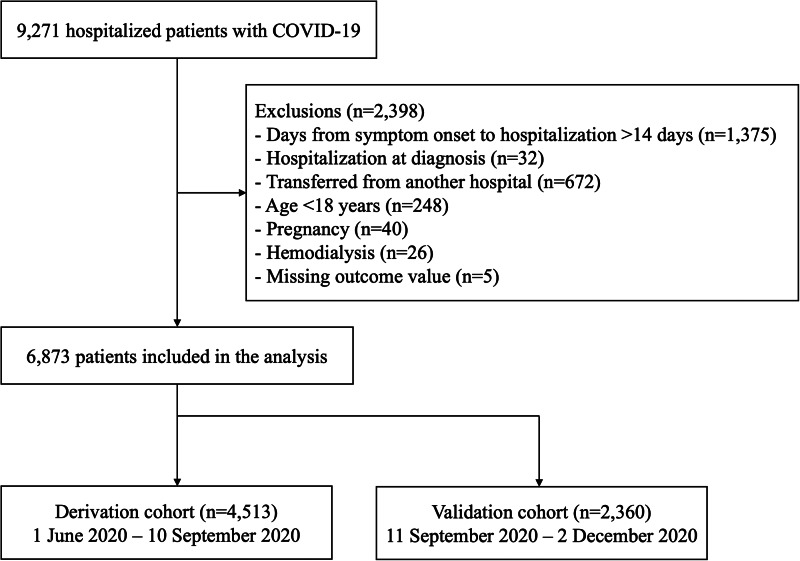
Flow chart of study participants, derivation cohort and validation cohort.

A total of 9271 patients tested positive for SARS-CoV-2 PCR during the study period, and 6873 patients were included in the final analysis, excluding those who met the exclusion criteria (Fig. 1). For the 4513 patients in the derivation cohort, median time from onset to admission was 4 days, 96.2% were Japanese, 58.8% were male, mean age was 46.9 years (s.d. = 20.1) and 20.7% required oxygen therapy (Table 1).

**Table 1.** Patient characteristics on admission

	Derivation cohort				Validation cohort			
		Overall	No oxygen	Oxygen		Overall	No oxygen	Oxygen
	% missing	*n* = 4513	*n* = 3578	*n* = 935	% missing	*n* = 2360	*n* = 1799	*n* = 561
Days from onset, mean (s.d.)	0	4.8 (2.9)	4.8 (2.9)	5.0 (3.0)	0	4.5 (3.0)	4.4 (3.0)	4.8 (3.0)
Days from onset, median (IQR)	0	4 (3–7)	4 (3–7)	5 (3–7)	0	4 (2–6)	4 (2–6)	4 (2–7)
Age (years), continuous, mean (s.d.)	0	46.9 (20.1)	42.3 (18.3)	64.5 (16.5)	0	50.7 (19.2)	46.5 (18.5)	64.3 (14.8)
Age (years), category, *n* (%)	0				0			
18–39		1891 (41.9)	1821 (50.9)	70 (7.5)		733 (31.1)	705 (39.2)	28 (5.0)
40–64		1615 (35.8)	1240 (34.7)	375 (40.1)		998 (42.3)	756 (42.0)	242 (43.1)
≥65		1007 (22.3)	517 (14.4)	490 (52.4)		629 (26.7)	338 (18.8)	291 (51.9)
Sex (male), *n* (%)	0.07	2651 (58.8)	2039 (57.0)	612 (65.5)	0.04	1398 (59.3)	1002 (55.7)	396 (70.6)
BMI (kg/m^2^), continuous	18.3	24.0 (5.2)	23.5 (5.2)	25.9 (5.1)	15.0	24.3 (5.3)	23.9 (5.4)	25.8 (4.8)
BMI (kg/m^2^), category	18.3				15.0			
<18.5		285 (7.7)	252 (8.6)	33 (4.4)		140 (7.0)	126 (8.2)	14 (3.0)
18.5–22.9		1471 (39.9)	1284 (43.7)	187 (24.9)		748 (37.3)	634 (41.3)	114 (24.1)
23.0–24.9		670 (18.2)	543 (18.5)	127 (16.9)		362 (18.0)	261 (17.0)	101 (21.4)
25.0–29.9		894 (24.3)	620 (21.1)	274 (36.5)		552 (27.5)	376 (24.5)	176 (37.2)
≥30.0		366 (9.9)	236 (8.0)	130 (17.3)		205 (10.2)	137 (8.9)	68 (14.4)
Ethnicity, *n* (%)	0.3				0.1			
Japanese		4327 (96.2)	3416 (95.8)	911 (97.7)		2213 (93.9)	1666 (92.7)	547 (97.9)
Asian		115 (2.6)	102 (2.9)	13 (1.4)		92 (3.9)	85 (4.7)	7 (1.3)
White		10 (0.2)	8 (0.2)	2 (0.2)		4 (0.2)	4 (0.2)	0 (0)
Black		8 (0.2)	8 (0.2)	0 (0)		4 (0.2)	4 (0.2)	0 (0)
Other		39 (0.9)	33 (0.9)	6 (0.6)		44 (1.9)	39 (2.2)	5 (0.9)
Smoking, *n* (%)	12.3				12.9			
Current		990 (25.0)	863 (27.4)	127 (15.9)		489 (23.8)	411 (26.0)	78 (16.5)
Former		823 (20.8)	546 (17.3)	277 (34.6)		499 (24.3)	323 (20.4)	176 (37.3)
Never		2143 (54.2)	1746 (55.3)	397 (49.6)		1067 (51.9)	849 (53.6)	218 (46.2)
Physician-diagnosed obesity, *n* (%)	0	253 (5.6)	150 (4.2)	103 (11.0)		145 (6.1)	102 (5.7)	43 (7.7)
Hypertension, *n* (%)	0	886 (19.6)	461 (12.9)	425 (45.5)	0	554 (23.5)	312 (17.3)	242 (43.1)
Hyperlipidaemia, *n* (%)	0	449 (9.9)	238 (6.7)	211 (22.6)	0	281 (11.9)	164 (9.1)	117 (20.9)
Diabetes, *n* (%)	0	465 (10.3)	225 (6.3)	240 (25.7)	0	279 (11.8)	131 (7.3)	148 (26.4)
Myocardial infarction, *n* (%)	0	54 (1.2)	29 (0.8)	25 (2.7)	0	30 (1.3)	9 (0.5)	21 (3.7)
Congestive heart failure, *n* (%)	0	59 (1.3)	20 (0.6)	39 (4.2)	0	26 (1.1)	12 (0.7)	14 (2.5)
Peripheral vascular disease, *n* (%)	0	27 (0.6)	13 (0.4)	14 (1.5)	0	18 (0.8)	9 (0.5)	9 (1.6)
Cerebrovascular disease, *n* (%)	0	129 (2.9)	60 (1.7)	69 (7.4)	0	77 (3.3)	39 (2.2)	38 (6.8)
Hemiplegia, *n* (%)	0	32 (0.7)	17 (0.5)	15 (1.6)	0	12 (0.5)	9 (0.5)	3 (0.5)
Dementia, *n* (%)	0	153 (3.4)	81 (2.3)	72 (7.7)	0	71 (3.0)	40 (2.2)	31 (5.5)
Asthma, *n* (%)	0	246 (5.5)	187 (5.2)	59 (6.3)	0	124 (5.3)	95 (5.3)	29 (5.2)
Chronic lung disease, *n* (%)	0	105 (2.3)	44 (1.2)	61 (6.5)	0	49 (2.1)	20 (1.1)	29 (5.2)
Chronic liver disease, *n* (%)	0	83 (1.8)	48 (1.3)	35 (3.7)	0	46 (1.9)	31 (1.7)	15 (2.7)
Peptic ulcer, *n* (%)	0	25 (0.6)	17 (0.5)	8 (0.9)	0	14 (0.6)	10 (0.6)	4 (0.7)
Moderate-to-severe renal dysfunction, *n* (%)	0	18 (0.4)	7 (0.2)	11 (1.2)	0	14 (0.6)	3 (0.2)	11 (2.0)
Malignancy, *n* (%)	0	104 (2.3)	56 (1.6)	48 (5.1)	0	70 (3.0)	45 (2.5)	25 (4.5)
Collagen disease, *n* (%)	0	35 (0.8)	24 (0.7)	11 (1.2)	0	30 (1.3)	19 (1.1)	11 (2.0)
HIV, *n* (%)	0	16 (0.4)	16 (0.4)	0 (0)	0	3 (0.1)	2 (0.1)	1 (0.2)
Immunosuppression, *n* (%)	0	70 (1.6)	42 (1.2)	28 (3.0)	0	44 (1.9)	28 (1.6)	16 (2.9)
Fever, *n* (%)	0.4	2090 (46.5)	1423 (39.9)	667 (71.9)	0.5	1107 (47.2)	729 (40.7)	378 (68.0)
Cough, *n* (%)	1.1	2250 (50.4)	1666 (47.0)	584 (63.5)	1.2	1270 (54.5)	937 (52.7)	333 (60.2)
Shortness of breath, *n* (%)	2.6	805 (18.3)	447 (12.8)	358 (39.5)	2.9	399 (17.4)	213 (12.2)	186 (34.4)
Wheezing	4.7	53 (1.2)	27 (0.8)	26 (3.0)	2.7	27 (1.2)	9 (0.5)	18 (3.4)
Fatigue	4.7	1760 (40.9)	1272 (37.0)	488 (56.2)	5.6	935 (42.0)	642 (37.8)	293 (55.3)

s.d., standard deviation; IQR, interquartile range; BMI, body mass index; HIV, human immunodeficiency virus.

Malignant disease was defined as the presence of solid cancer, leukaemia, lymphoma or metastatic solid cancer. Immunosuppressed status was defined as neutrophil count <500/μl, use of glucocorticoids within the past month, chemotherapy within the past 3 months, radiotherapy within the past 3 months, use of immunosuppressive drugs within the past 3 months, blood transplant, organ transplant, asplenic syndrome or primary immunodeficiency.

### Model development

Odds ratios (ORs), 95% confidence intervals and *P*-values for each predictor as calculated by multivariable logistic regression analysis for each age group, as well as the comprehensive model and the simple risk score model calculated from it, are shown in Tables 2–4 and Supplementary Table S2a–c. Variables selected for each age group differed, with seven variables (sex, age, BMI, malignancy, fever, SOB and wheezing) in the young age group (18–39 years) and eight variables (sex, age, BMI, diabetes, fever, cough, SOB and fatigue) in the middle-aged group. In the elderly, nine variables (age, BMI, congestive heart failure, cerebrovascular disease, diabetes, hypertension, fever, cough and SOB) were selected.

**Table 2.** Multivariable analysis in patients 18–39 years old

Derivation cohort				Comprehensive model	Simple risk score
	Odds ratio	95% CI	*P*	Coefficient	Points
Male	2.83	1.34–6.01	0.007	1.04	1
Age ≥30 years	3.46	1.90–6.28	<0.001	1.24	1
Body mass index					
<18.5 kg/m^2^	0.68	0.09–5.41	0.715	−0.39	0
18.5–22.9 kg/m^2^	Reference				
23.0–24.9 kg/m^2^	1.93	0.80–4.70	0.146	0.66	1
25.0–29.9 kg/m^2^	2.08	0.96–4.52	0.064	0.73	1
≥30.0 kg/m^2^	6.85	3.18–14.78	<0.001	1.92	2
Malignancy	13.58	1.33–138.46	0.028	2.61	3
Fever	4.83	2.54–9.19	<0.001	1.57	2
Shortness of breath	3.60	2.03–6.39	<0.001	1.28	1
Wheezing	5.57	1.22–25.40	0.027	1.72	2
Intercept			<0.001	−6.68

**Table 3.** Multivariable analysis in patients 40–64 years old

Derivation cohort				Comprehensive model	Simple risk score
	Odds ratio	95% CI	*P*	Coefficient	Points
Male	1.67	1.23–2.28	0.001	0.51	1
Age					
40–49 years	Reference				
50–59 years	1.61	1.20–2.16	0.002	0.48	1
60–64 years	3.62	2.49–5.27	<0.001	1.29	3
Body mass index					
<18.5 kg/m^2^	1.01	0.42–2.42		0.01	0
18.5–22.9 kg/m^2^	Reference				
23.0–24.9 kg/m^2^	1.17	0.77–1.79	0.457	0.16	0
25.0–29.9 kg/m^2^	2.21	1.54–3.17	<0.001	0.79	2
≥30.0 kg/m^2^	2.57	1.69–3.91	<0.001	0.94	2
Diabetes	1.72	1.21–2.45	0.003	0.54	1
Fever	2.18	1.64–2.88	<0.001	0.78	2
Cough	1.85	1.40–2.45	<0.001	0.61	1
Shortness of breath	2.90	2.17–3.88	<0.001	1.07	2
Fatigue	1.65	1.26–2.16	<0.001	0.50	1
Intercept				−3.92

**Table 4.** Multivariable analysis in patients ≥65 years old

Derivation cohort				Comprehensive model	Simple risk score
	Odds ratio	95% CI	*P*	Coefficient	Points
Age ≥75 years	1.98	1.47–2.67	<0.001	0.68	2
BMI					
<18.5 kg/m^2^	0.89	0.51–1.54	0.671	−0.12	0
18.5–22.9 kg/m^2^	Reference				
23.0–24.9 kg/m^2^	1.00	0.68–1.48	0.999	0.00	0
25.0–29.9 kg/m^2^	2.02	1.38–2.94	<0.001	0.70	2
≥30.0 kg/m^2^	1.77	0.96–3.27	0.069	0.57	2
Congestive heart failure	2.13	1.04–4.36	0.039	0.76	2
Cerebrovascular disease	1.66	1.02–2.68	0.040	0.50	1
Diabetes	2.02	1.41–2.89	<0.001	0.70	2
Hypertension	1.80	1.34–2.43	<0.001	0.59	2
Fever	3.58	2.67–4.80	<0.001	1.28	4
Cough	1.42	1.06–1.90	0.020	0.35	1
Shortness of breath	4.67	3.20–6.80	<0.001	1.54	4
Intercept				−2.32	

### Model validation

**Fig. 2.**
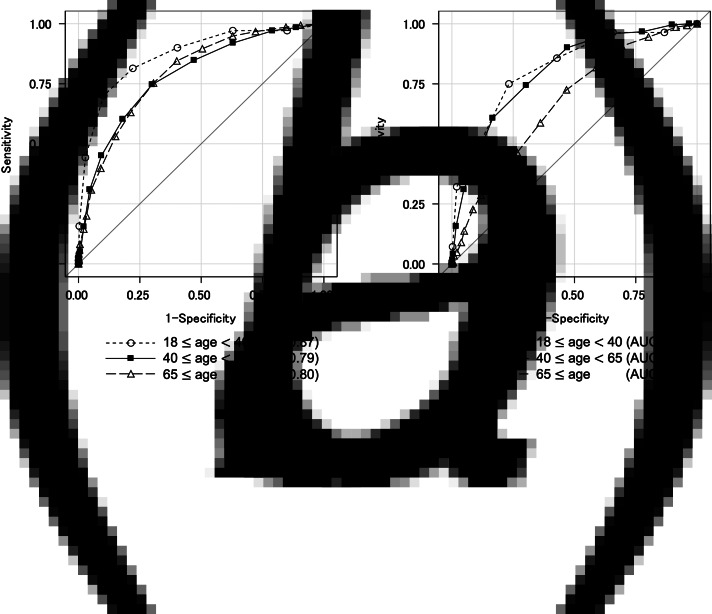
Receiver operator characteristic curves for simple risk score model of each age group. (a) Derivation cohort; (b) validation cohort.

**Fig. 3.**
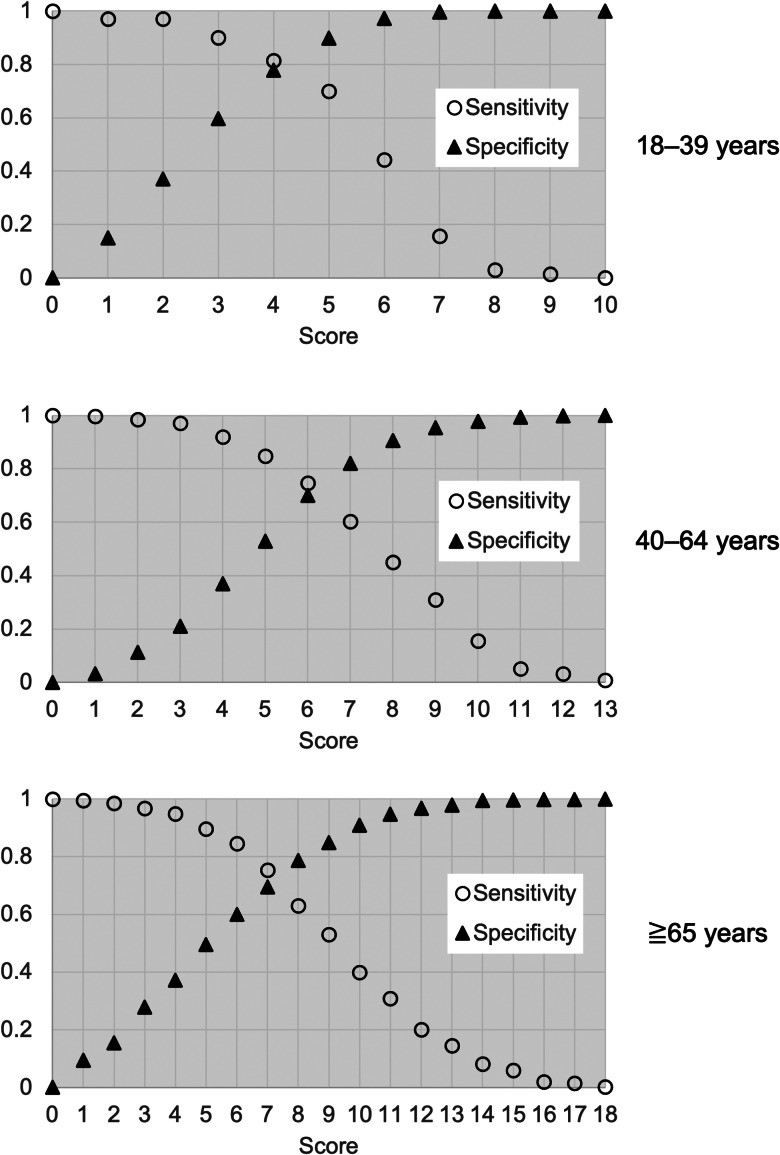
Sensitivity and specificity of the models for each age group at various cut-offs to detect respiratory failure.

**Fig. 4.**
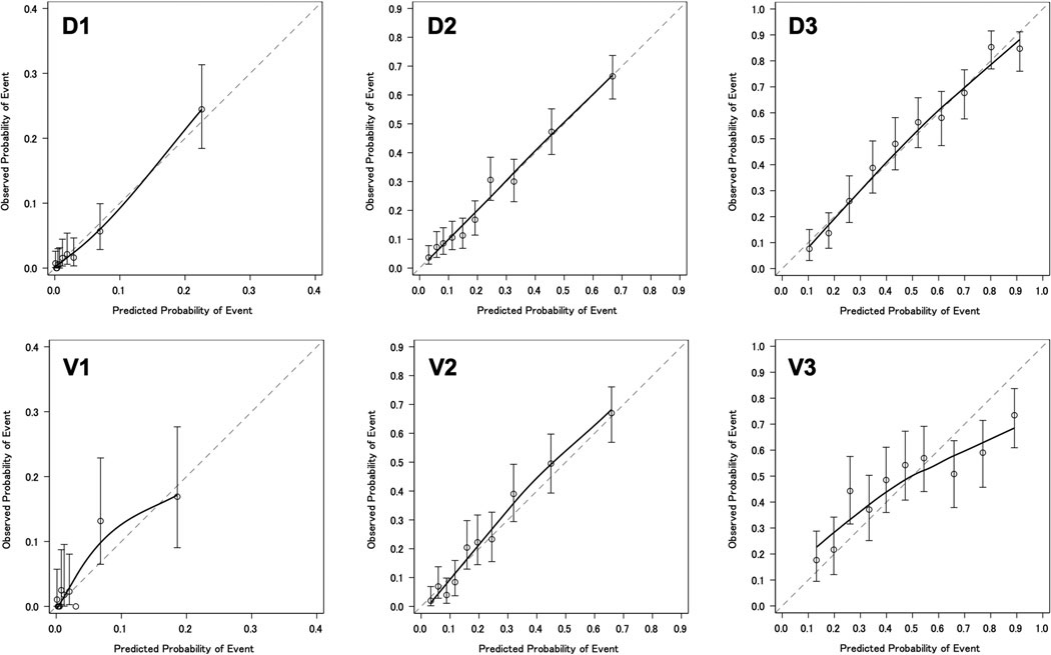
Calibration plot of the comprehensive model. Derivation cohort (D1, 18–39 years; D2, 40–64 years; D3, ≥65 years) and validation cohort (V1, 18–39 years; V2, 40–64 years; V3, ≥65 years).

In the derivation cohort, AUCs of the simple risk score models were 0.87, 0.79 and 0.80 in the young, middle-aged and elderly groups, respectively. In the validation cohort, AUCs from simple risk score models were 0.81, 0.80 and 0.67 in the young, middle-aged and elderly groups, respectively (Fig. 2). Figure 3 and Supplementary Table S3 show the sensitivity, specificity, PPV and NPV of models for each age group at various cut-offs. Figure 4 shows calibration plots for the derivation cohort and validation cohort. In the sensitivity analysis, the models obtained by the analysis of imputed data, when missing BMI data were complemented according to the distribution of observed BMIs, were not significantly different from those obtained by primary analysis when missing BMI data were imputed using the regression method (Supplementary Table S4 and Fig. S1).

## Discussion

This study developed a simple score to predict the need for oxygen therapy among COVID-19 patients using age, sex, BMI, underlying diseases, and symptoms for young, middle-aged and elderly age groups. We found that although BMI and SOB were factors with relatively high impact in all age groups, the models selected for each age group were quite different.

Although many predictive models for COVID-19 have been developed [11], the novelty of this study lies in the development of a model that predicts oxygen demand by age group using only the underlying diseases of the patient and symptom data from a large dataset.

Among the prediction models developed in this study, BMI, diabetes mellitus, hypertension, congestive heart failure, cerebrovascular disease, malignancy, fever and cough have been selected in previous studies [16, 30–34] and were consistent with previous results.

PCR testing has become widely available, and COVID-19 has been diagnosed in general clinics and testing centres. In addition, telemedicine and telephone follow-up have increased since the start of the COVID-19 pandemic [35]. In this situation, using information such as blood sampling data, physical examinations and oxygen saturation is difficult. Therefore, the models in this study appear useful to predict patients likely to experience respiratory failure in the future and to support the decision-making of healthcare providers. COVID-19 patients have been pointed out to be potentially unaware of dyspnoea even if they are hypoxic [36, 37]. The present models may also be clinically applicable to patients with mild or asymptomatic disease who are observed at home or in an institution to closely monitor high-risk patients. In addition, under situations such as a pandemic, where the workload on healthcare workers and health centre staff is high, a simple model that can be calculated using only a pen and paper is more convenient than a complicated predictive model that requires an online computer.

The use of this score in the field might vary depending on the local COVID-19 caseload. For example, it can be used as a criterion to determine the indication for hospitalisation in situations where many patients require oxygenation, or when the number of cases is low, it can be used to select the appropriate healthcare-related facilities for patients with differing levels of disease severity.

We propose that the cut-off value for the simple score model of this study should be ≥6 for the model of young people (18–39 years old), ≥5 for the model of middle-aged people (40–64 years old) and ≥3 for the model of elderly people (≥65 years old). The reasons are as follows. In the younger population, fewer patients progress to respiratory failure, so lowering the cut-off will lower the PPV and increase unnecessary hospitalisations. In the younger population, where remaining physical capacity is usually much greater, selection of high-risk patients for inpatient care or intensive follow-up is preferable. In the middle-aged population, where about one-third of patients develop respiratory failure, a cut-off of ≥5 provides 85% sensitivity, and nearly half of the hospitalised patients who do not require oxygen can be classified as low-risk. In the elderly population, about half develop respiratory failure during hospitalisation. For the elderly, it is important not to overlook those who would suffer from respiratory failure, and the cut-off should thus be set as low as possible. A cut-off value of ≥3 provides 97% sensitivity, a PPV of 56% and an NPV of 90%, representing a reasonable criterion for making decisions about hospitalisation for this disease with a high mortality rate.

### Limitations

Various limitations to this investigation need to be considered when interpreting the results. This study was analysed using registry data from COVID-19 patients hospitalised in Japan. The population used to develop the model is thus expected to be sicker than the overall population of COVID-19 patients, including those who have not been hospitalised. However, in Japan, COVID-19 patients ≥65 years old and patients <65 years old with underlying diseases were recommended to be hospitalised in principle. This study cohort may thus offer a relatively good reflection of the COVID-19 population. In the younger age group (18–39 years), the effects of predictor variables may not have been properly assessed because of the small number of patients in whom the endpoint occurred. When using this prediction model in clinical practice, it is important to note that the endpoint used in this study was the presence or absence of oxygen therapy. Patients may require hospitalisation for supportive care for medical conditions other than respiratory failure, such as dehydration or poor food intake. Therefore, even if a patient gives a negative result under the prediction model described in this study, follow-up of the patient based on the clinical situation is warranted. The performance of the prediction model may change in the future with the extensive use of vaccines and the spread of viral mutations. Since most patients analysed were ethnically Japanese, the validity of the model in other populations needs to be verified. It should be noted that some patients who are considered to have no underlying medical conditions may actually have a high risk, as they may not be aware of their own health status or may not have visited a medical institution for a long period of time. Finally, the performance of the model for elderly people (≥65 years old) decreased when applied to the validation cohort. A possible reason for this may be that we have a lot of cluster cases at nursing homes included in the validation cohort, so that the characteristics of elderly people in the validation cohort may have been much different from the characteristics of those in the derivation cohort [38]. Therefore, further studies to update the model are needed.

## Conclusion

Using data from the large COVID-19 hospitalisation registry in Japan, we developed a simple predictive score model to predict respiratory failure based solely on the underlying diseases and symptoms of patients. A simple risk score based on readily available information is highly versatile and may be useful during pandemics.

## Data Availability

No data are available.

## Supplementary material

For supplementary material accompanying this paper visit http://dx.doi.org/10.1017/S0950268821001837. click here to view supplementary material
