# Supplementary material for: Predicting respiratory failure for COVID-19 patients in Japan: a simple clinical score for evaluating the need for hospitalisation
Source: Epidemiol Infect. 2021 Jul 30;149:e175. doi: 10.1017/S0950268821001837 (PMC8365048; doi:10.1017/S0950268821001837)
Supplement: Supplementary file 1 [file hygsup.zip › S0950268821001837sup002.docx]

*Epidemiology and Infection*

Predicting respiratory failure for COVID-19 patients in Japan: a simple clinical score for evaluating the need for hospitalization

Supplementary Table S1.

| Variables | Definitions |
| --- | --- |
| **Comorbidities** |  |
| Congestive heart failure | Physician-diagnosed or self-reported |
| Cerebrovascular disease | Physician-diagnosed or self-reported |
| Chronic lung disease | Physician-diagnosed or self-reported, excluding asthma |
| Asthma | Physician-diagnosed or self-reported |
| Chronic liver disease | Physician-diagnosed or self-reported, including liver cirrhosis |
| Diabetes | Physician-diagnosed or self-reported, with or without complication |
| Hypertension | Physician-diagnosed or self-reported |
| Hyperlipidemia | Physician-diagnosed or self-reported |
| Moderate-to-severe renal dysfunction | Physician-diagnosed or self-reported, serum creatinine $\geq$ 3 mg/dL |
| Malignancy | Physician-diagnosed or self-reported, including solid tumour, leukemia, lymphoma, metastatic solid tumour within 5 years |
| Collagen disease | Physician-diagnosed or self-reported |
| **Symptoms** |  |
| Fever | Self-reported |
| Cough | Self-reported |
| Shortness of breath | Self-reported |
| Wheezing | Self-reported |
| Fatigue | Self-reported |

Supplementary Table S2a. Multivariable analysis in patients 18–39 years old

| Age 18–39 years | | |
| --- | --- | --- |
| Variables | Comprehensive model | Simple risk score |
| Male | 1.04 | 1 |
| Age≧30 years | 1.24 | 1 |
| Body mass index |  |  |
| <18.5 kg/m^2^ | -0.39 | 0 |
| 18.5–22.9 kg/m^2^ | Reference | 0 |
| 23.0–24.9 kg/m^2^ | 0.66 | 1 |
| 25.0–29.9 kg/m^2^ | 0.73 | 1 |
| ≧30.0 kg/m^2^ | 1.92 | 2 |
| Congestive heart failure |  | 0 |
| Cerebrovascular disease |  | 0 |
| Chronic lung disease |  | 0 |
| Asthma |  | 0 |
| Chronic liver disease |  | 0 |
| Diabetes |  | 0 |
| Hypertension |  | 0 |
| Hyperlipidemia |  | 0 |
| Moderate-to-severe renal dysfunction |  | 0 |
| Malignancy | 2.61 | 3 |
| Collagen disease |  | 0 |
| Fever | 1.57 | 2 |
| Cough |  | 0 |
| Shortness of breath | 1.28 | 1 |
| Wheezing | 1.72 | 2 |
| Fatigue |  | 0 |
| Intercept | -6.68 |  |

Supplementary Table S2b. Multivariable analysis in patients 40–64 years old

| Age 40–64 years | | |
| --- | --- | --- |
| Variables | Comprehensive model | Simple risk score |
| Male | 0.51 | 1 |
| Age |  |  |
| 40–49 years | Reference | 0 |
| 50–59 years | 0.48 | 1 |
| 60–64 years | 1.29 | 3 |
| Body mass index |  |  |
| <18.5 kg/m^2^ | 0.01 | 0 |
| 18.5–22.9 kg/m^2^ | Reference | 0 |
| 23.0–24.9 kg/m^2^ | 0.16 | 0 |
| 25.0–29.9 kg/m^2^ | 0.79 | 2 |
| ≧30.0 kg/m^2^ | 0.94 | 2 |
| Congestive heart failure |  | 0 |
| Cerebrovascular disease |  | 0 |
| Chronic lung disease |  | 0 |
| Asthma |  | 0 |
| Chronic liver disease |  | 0 |
| Diabetes | 0.54 | 1 |
| Hypertension |  | 0 |
| Hyperlipidemia |  | 0 |
| Moderate-to-severe renal dysfunction |  | 0 |
| Malignancy |  | 0 |
| Collagen disease |  | 0 |
| Fever | 0.78 | 2 |
| Cough | 0.61 | 1 |
| Shortness of breath | 1.07 | 2 |
| Wheezing |  | 0 |
| Fatigue | 0.50 | 1 |
| Intercept | -3.92 |  |

Supplementary Table S2c. Multivariable analysis in patients ≥65 years old

| Age ≧65 years | | |
| --- | --- | --- |
| Variables | Comprehensive model | Simple risk score |
| Male |  | 0 |
| Age≧75 years | 0.68 | 2 |
| Body mass index |  | 0 |
| <18.5 kg/m^2^ | -0.12 | 0 |
| 18.5–22.9 kg/m^2^ | Reference | 0 |
| 23.0–24.9 kg/m^2^ | 0.00 | 0 |
| 25.0–29.9 kg/m^2^ | 0.70 | 2 |
| ≧30.0 kg/m^2^ | 0.57 | 2 |
| Congestive heart failure | 0.76 | 2 |
| Cerebrovascular disease | 0.50 | 1 |
| Chronic lung disease |  | 0 |
| Asthma |  | 0 |
| Chronic liver disease |  | 0 |
| Diabetes | 0.70 | 2 |
| Hypertension | 0.59 | 2 |
| Hyperlipidemia |  | 0 |
| Moderate-to-severe renal dysfunction |  | 0 |
| Malignancy |  | 0 |
| Collagen disease |  | 0 |
| Fever | 1.28 | 4 |
| Cough | 0.35 | 1 |
| Shortness of breath | 1.54 | 4 |
| Wheezing |  | 0 |
| Fatigue |  | 0 |
| Intercept | -2.32 | 0 |

Supplementary Table S3. Sensitivity, specificity, positive predictive value, and negative predictive value of the simple risk score model at different cutoffs

|  |  | Derivation cohort | | | |  | Validation cohort | | | |
| --- | --- | --- | --- | --- | --- | --- | --- | --- | --- | --- |
|  | Cutoff | Sens | Spec | PPV | NPV |  | Sens | Spec | PPV | NPV |
| 18–39 years | 1 | 0.971 | 0.371 | 0.042 | 0.993 |  | 0.964 | 0.133 | 0.042 | 0.990 |
|  | 2 | 0.971 | 0.150 | 0.056 | 0.997 |  | 0.964 | 0.343 | 0.055 | 0.996 |
|  | 3 | 0.900 | 0.597 | 0.079 | 0.994 |  | 0.857 | 0.572 | 0.074 | 0.990 |
|  | 4 | 0.814 | 0.779 | 0.124 | 0.991 |  | 0.750 | 0.767 | 0.114 | 0.987 |
|  | 5 | 0.700 | 0.898 | 0.209 | 0.987 |  | 0.464 | 0.904 | 0.161 | 0.977 |
|  | 6 | 0.443 | 0.972 | 0.378 | 0.978 |  | 0.321 | 0.980 | 0.391 | 0.973 |
|  |  |  |  |  |  |  |  |  |  |  |
|  | Cutoff | Sens | Spec | PPV | NPV |  | Sens | Spec | PPV | NPV |
| 40–64 years | 3 | 0.971 | 0.210 | 0.271 | 0.959 |  | 0.967 | 0.224 | 0.285 | 0.955 |
|  | 4 | 0.920 | 0.370 | 0.306 | 0.939 |  | 0.959 | 0.357 | 0.323 | 0.964 |
|  | 5 | 0.848 | 0.529 | 0.353 | 0.920 |  | 0.901 | 0.529 | 0.380 | 0.943 |
|  | 6 | 0.747 | 0.700 | 0.429 | 0.901 |  | 0.744 | 0.697 | 0.440 | 0.895 |
|  | 7 | 0.603 | 0.819 | 0.502 | 0.872 |  | 0.607 | 0.833 | 0.539 | 0.869 |
|  | 8 | 0.451 | 0.906 | 0.593 | 0.845 |  | 0.417 | 0.909 | 0.594 | 0.830 |
|  |  |  |  |  |  |  |  |  |  |  |
|  | Cutoff | Sens | Spec | PPV | NPV |  | Sens | Spec | PPV | NPV |
| ≧65 years | 3 | 0.967 | 0.279 | 0.560 | 0.900 |  | 0.945 | 0.198 | 0.504 | 0.807 |
|  | 4 | 0.949 | 0.371 | 0.589 | 0.885 |  | 0.907 | 0.308 | 0.530 | 0.794 |
|  | 5 | 0.896 | 0.495 | 0.627 | 0.834 |  | 0.818 | 0.411 | 0.545 | 0.724 |
|  | 6 | 0.845 | 0.600 | 0.667 | 0.803 |  | 0.725 | 0.533 | 0.572 | 0.692 |
|  | 7 | 0.753 | 0.694 | 0.700 | 0.748 |  | 0.588 | 0.639 | 0.584 | 0.643 |
|  | 8 | 0.631 | 0.787 | 0.738 | 0.692 |  | 0.464 | 0.734 | 0.600 | 0.614 |

Abbreviations: Sens, sensitivity; Spec, specificity; PPV, positive predictive value; NPV, negative predictive value

Supplementary Table S4. Comprehensive model and simple risk score model when missing BMI data were complemented according to the distribution of observed BMIs.

| Age 18–39 years | | |  | Age 40–64 years | | |  | Age ≧65 years | | |
| --- | --- | --- | --- | --- | --- | --- | --- | --- | --- | --- |
| Variables | Compre-hensive model | Simple risk score |  | Variables | Compre-hensive model | Simple risk score |  | Variables | Compre-hensive model | Simple risk score |
| Male | 1.10 | 1 |  | Male | 0.53 | 1 |  | Age≧75 years | 0.70 | 2 |
| Age≧30 years | 1.24 | 1 |  | Age |  |  |  | Body mass index |  |  |
| Body mass index |  |  |  | 40–49 years |  |  |  | <18.5 kg/m^2^ | 0.22 | 1 |
| <18.5 kg/m^2^ | 0.43 | 0 |  | 50–59 years | 0.49 | 1 |  | 18.5–22.9 kg/m^2^ |  |  |
| 18.5–22.9 kg/m^2^ |  |  |  | 60–64 years | 1.28 | 2 |  | 23.0–24.9 kg/m^2^ | -0.16 | 0 |
| 23.0–24.9 kg/m^2^ | 0.52 | 0 |  | Body mass index |  |  |  | 25.0–29.9 kg/m^2^ | 0.49 | 1 |
| 25.0–29.9 kg/m^2^ | 0.84 | 1 |  | <18.5 kg/m^2^ | -0.47 | -1 |  | ≧30.0 kg/m^2^ | 0.43 | 1 |
| ≧30.0 kg/m^2^ | 1.97 | 2 |  | 18.5–22.9 kg/m^2^ |  |  |  | Diabetes | 0.78 | 2 |
| Malignancy | 2.45 | 2 |  | 23.0–24.9 kg/m^2^ | 0.10 | 0 |  | Hypertension | 0.68 | 2 |
| Fever | 1.64 | 1 |  | 25.0–29.9 kg/m^2^ | 0.65 | 1 |  | Fever | 1.27 | 3 |
| Shortness of breath | 1.33 | 1 |  | ≧30.0 kg/m^2^ | 0.97 | 2 |  | Cough | 0.37 | 1 |
| Wheezing | 1.71 | 2 |  | Diabetes | 0.54 | 1 |  | Shortness of breath | 1.55 | 4 |
| Intercept | -6.82 |  |  | Fever | 0.78 | 2 |  | Intercept |  |  |
|  |  |  |  | Cough | 0.62 | 1 |  |  |  |  |
|  |  |  |  | Shortness of breath | 1.05 | 2 |  |  |  |  |
|  |  |  |  | Fatigue | 0.51 | 1 |  |  |  |  |
|  |  |  |  | Intercept | -3.86 |  |  |  |  |  |
