# Supplementary figures and images for: Predicting respiratory failure for COVID-19 patients in Japan: a simple clinical score for evaluating the need for hospitalisation
Source: Epidemiol Infect. 2021 Jul 30;149:e175. doi: 10.1017/S0950268821001837 (PMC8365048; doi:10.1017/S0950268821001837)

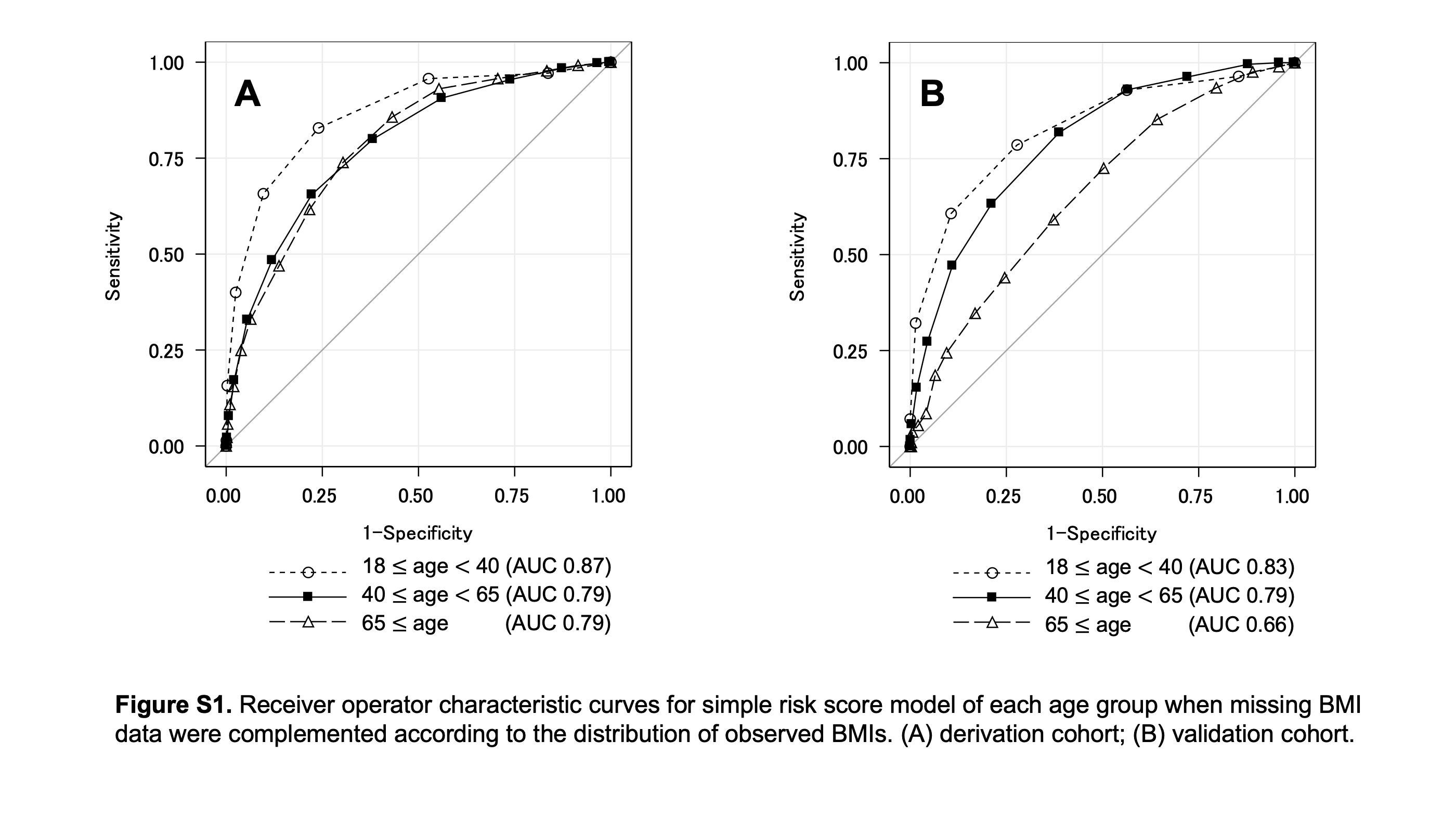

Supplement: Supplementary file 1 [file hygsup.zip › S0950268821001837sup001.tiff]
